# Insight into the specific virulence related genes and toxin-antitoxin virulent pathogenicity islands in swine streptococcosis pathogen *Streptococcus equi* ssp. *zooepidemicus* strain ATCC35246

**DOI:** 10.1186/1471-2164-14-377

**Published:** 2013-06-07

**Authors:** Jianing Geng, Li Yi, Bin Xu, Ruoyu Jia, Yue Li, Qingshu Meng, Hongjie Fan, Songnian Hu

**Affiliations:** 1 College of Veterinary Medicine, Nanjing Agricultural University, Nanjing 210095, People’s Republic of China; 2 CAS Key Laboratory of Genome Sciences and Information, Beijing Institute of Genomics, Chinese Academy of Sciences, Beijing, China

## Abstract

**Background:**

*Streptococcus equi* ssp. *zooepidemicus* (*S*. z*ooepidemicus*) is an important pathogen causing swine streptococcosis in China. Pathogenicity islands (PAIs) of *S*. z*ooepidemicus* have been transferred among bacteria through horizontal gene transfer (HGT) and play important roles in the adaptation and increased virulence of *S*. z*ooepidemicus.* The present study used comparative genomics to examine the different pathogenicities of *S*. z*ooepidemicus*.

**Results:**

Genome of *S. zooepidemicus* ATCC35246 (Sz35246) comprises 2,167,264-bp of a single circular chromosome, with a GC content of 41.65%. Comparative genome analysis of Sz35246, *S. zooepidemicus* MGCS10565 (Sz10565), *Streptococcus equi.* ssp. e*qui.* 4047 (Se4047) and *S. zooepidemicus* H70 (Sz70) identified 320 Sz35246-specific genes, clustered into three toxin-antitoxin (TA) systems PAIs and one restriction modification system (RM system) PAI. These four acquired PAIs encode proteins that may contribute to the overall pathogenic capacity and fitness of this bacterium to adapt to different hosts. Analysis of the in vivo and in vitro transcriptomes of this bacterium revealed differentially expressed PAI genes and non-PAI genes, suggesting that Sz35246 possess mechanisms for infecting animals and adapting to a wide range of host environments. Analysis of the genome identified potential Sz35246 virulence genes. Genes of the Fim III operon were presumed to be involved in breaking the host-restriction of Sz35246.

**Conclusion:**

Genome wide comparisons of Sz35246 with three other strains and transcriptome analysis revealed novel genes related to bacterial virulence and breaking the host-restriction. Four specific PAIs, which were judged to have been transferred into Sz35246 genome through HGT, were identified for the first time. Further analysis of the TA and RM systems in the PAIs will improve our understanding of the pathogenicity of this bacterium and could lead to the development of diagnostics and vaccines.

## Background

PAIs play important roles in the adaptation and increased virulence of pathogens. Bacterial PAI often encode both effector molecules responsible for disease and secretion systems that deliver these effectors to host cells [1,2]. PAIs are a distinct type of genomic island. PAIs contain mobile genetic elements (MGEs), which were acquired by the bacteria through HGT. Bacterial genomes contain various types of MGEs, such as transposons, plasmids, and bacteriophages. All of these elements may be acquired by HGT. Many MGEs serve as shuttles for genes that are beneficial to bacteria during their proliferation in a host environment. Several MGEs have been found in the genomes of pathogenic bacteria that contain genes conferring antibiotic resistance and genes encoding virulence factors, such as exotoxins, adhesins, and secretion systems [3].

Pathogenic bacteria often make use of suicide mechanisms, in which the death of individual cells benefits the survival of the population. This mechanism is regulated by the toxin-antitoxin system (TA system), which is related to DNA replication, mRNA stability, protein synthesis, cell-wall biosynthesis and ATP synthesis [4]. The ϵ antitoxin-ζ toxin system (ϵ/ζ system) is a type II TA system. It is distributed over plasmids and chromosomes of various pathogenic bacteria [5]. These systems benefit the stability of the genomic island in the bacterial genome.

*S. zooepidemicus* is the ancestor of *Streptococcus equi ssp. equi* (*S. equi*) and these two strains express many of the same proteins and virulence factors. However, unlike *S. equi*, which is host-restricted and only infects horses, *S. zooepidemicus* has no host preference. *S. zooepidemicus* is primarily an opportunistic pathogen infecting a wide variety of animal species, including important domestic species, which makes it a pathogen of veterinary concern. *S. zooepidemicus* causes mastitis in cows and mares, and is the most frequently isolated opportunistic pathogen of horses [6]. Occasionally, *S. zooepidemicus* can infect humans via zoonotic transmission from infected animals and causes invasive infections in humans such as septicemia and meningitis [7,8]. In 1975, Sichuan province experienced an *S. zooepidemicus* outbreak that resulted in the death of 300,000 pigs and great economic losses. *S. zooepidemicus* is an important pathogen of streptococcal diseases in swine [9,10] and it remains a great threat to Chinese swine breeding. In the present study, we used comparative genomic analyses between *S. zooepidemicus* ATCC35246 and other published *S. zooepidemicus* strains [11,12] to investigate the mechanisms underlying the differing pathogenicities of *Streptococcus equi ssp.* In particular, we tried to ascertain how *S. zooepidemicus* ATCC35246 is able to cause such a serious disease in pig. We determined the complete genome sequence of *S. zooepidemicus* ATCC35246 (Sz35246), a virulent strain isolated from a dead pig in China. The complete genome sequence not only permitted detailed analysis of the phylogenic relationship between species, but also provided insights into the biology and pathogenic capacity of this streptococcus.

## Results and discussion

### Genomic features and basic transcriptomic structure

**Figure 1:**
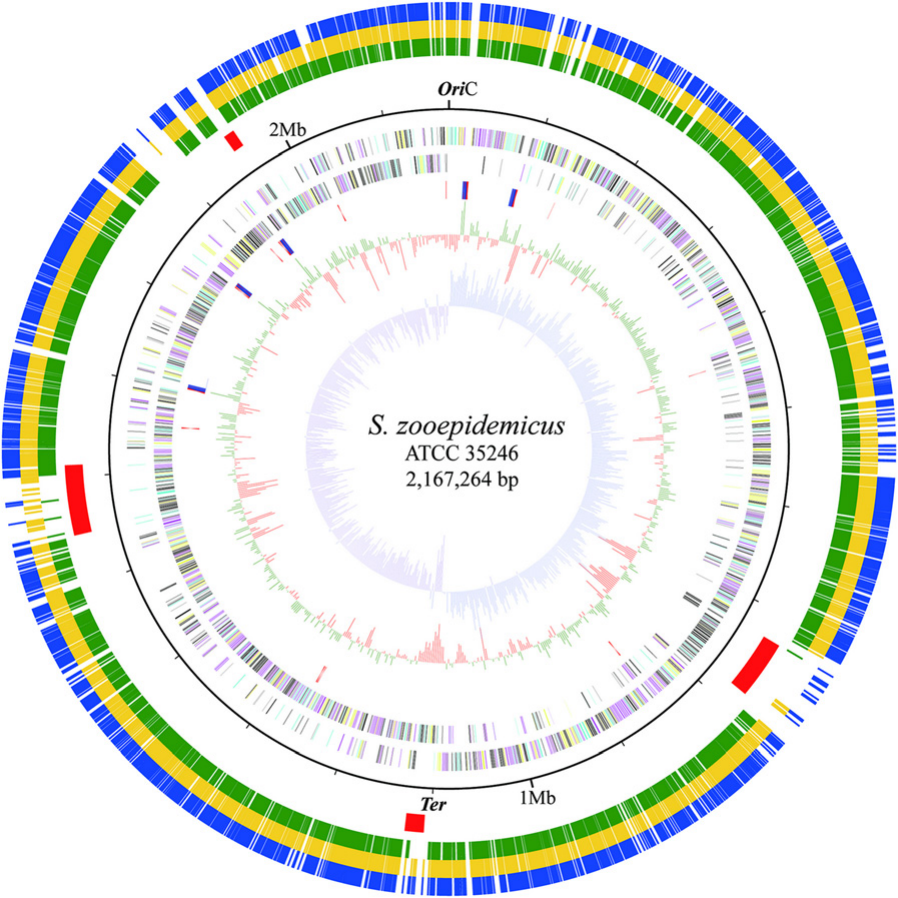
**Circular representation of the *S. zoopedemicus *ATCC35246 genome and comparative genome results.** The ten circles (outer to inner) show the following. The outer three circles represent Sz35246 protein-encoding genes homologous to that of Sz10565, Sz70 and Se4047, respectively. The fourth circle represents the PAI in Sz35246 chromosome. The fifth circle shows the chromosome position scaled in kb from *oriC*. The sixth and seventh circles show the coding sequences on the plus and minus strands, respectively. All genes are color-coded based on the COG functional categories: cyan, information storage and processing; yellow, cellular processes and signaling; magenta, metabolism; and black, poorly characterized. The eighth circle shows rRNA in red and tRNA in blue. The ninth circle shows the GC content (in 1-kb windows).Values that are greater than and or less than the average (41.65%) are shown in green and red, respectively. The tenth circle shows the GC skew curve (10-kb window and 1-kb incremental shift). The values for plus and minus strands are shown in cobalt blue and purple, respectively.

The 2,167,264-bp genome of Sz35246 comprises a single circular chromosome with a GC content of 41.65% (Additional file 1: Table S1 & Figure 1) and the genome information have been reported previously [11]. The GC content is similar to that of *Streptococcus equi* subsp. *zooepidemicus* MGCS10565 (Sz10565) [12], *Streptococcus equi* subsp. *equi* 4047 (Se4047) and *Streptococcus equi* subsp. *zooepidemicus* H70 (Sz70) [13]. The genome contains 2,087 protein-encoding genes, 57 tRNA genes, and five 5S-16S-23S rRNA operon gene clusters. Among the protein coding genes, 416 (19.93%) are predicted to encode conserved hypothetical proteins that are similar to proteins of unknown factions in other genomes, and 137 hypothetical genes (6.56%) have no matches in the nr protein database (Additional file 1: Table S1). The remaining 1534 genes were assigned putative functions. Eighty-one genes were identified as mobile elements, including those encoding a competence protein, a phage associated protein, a conjugation protein, a transposase and a site-specific recombinase, suggesting that these elements are used to take up and incorporate foreign DNA and are involved in reconstructing the genome architecture. Furthermore, global transcriptome analysis of Sz35246 using RNA-seq confirmed that 2048 of the 2,087 ORFs are expressed, but with different sequence coverages in vitro and in vivo (Additional file 1: Table S1). Comparative gene expression analysis reveals that 252 genes are upregulated and 142 genes are downregulated (Additional file 2: Table S2) by more than a 2-fold change in reads per kilo base per million (RPKM) values (p < 0.001) in vivo. The upregulated genes include 67 hypothetical protein coding genes and 28 response regulator, transcription regulator genes and chaperone protein encoding genes, suggesting that the differential expression of these ORFs plays an important role in survival of Sz35246 within the different host environments.

Additionally, we found that some genes, including *mal*A (SeseC_01626), *mal*D (SeseC_01627), *mal*E (SeseC_01633, SeseC_01622), *mal*F (SeseC_01624, SeseC_01630), *mal*G (SeseC_01625) and *mal*Q (SeseC_01617) were upregulated when Sz35246 infected mice. These genes are related to maltose transport and metabolism and utilization of carbohydrates, which is essential for the ability of pathogenic bacteria to cause disease. Group A *Streptococcus* (GAS) strains express *mal*E on their surface, and the transcript levels of the *mal*E gene were significantly increased during growth in human saliva compared to common medium. MalE may contribute to the ability of GAS to colonize the oropharynx by utilizing maltose [14]. In addition, studies in *S. pneumoniae* have shown that deletions in carbon metabolism genes, including the maltose operon, lead to decreased production of known virulence factors, such as capsular polysaccharide and cholera toxin [15]. MalE of Sz35246 is a maltodextrin-binding protein, which also binds longer maltodextrins (e.g., maltotriose and maltotetraose). The upregulation of this protein and other maltose utilization-related proteins may contribute to the infection of Sz35246. Further investigation into these carbohydrate transport and metabolism pathways genes may yield novel insights into the pathogenesis of Sz35246. We also observed that certain known virulence factors were upregulated during Sz35246 infection, for example, streptokinase (SeseC_02411) and fibronectin-binding protein (*sfs*, SeseC_00464). The upregulation of bacteriocin (SeseC_02042) could help Sz35246 compete with other bacteria that colonize the host.

### Comparative genomic analysis and pathogenicity islands (PAIs)

**Figure 2:**
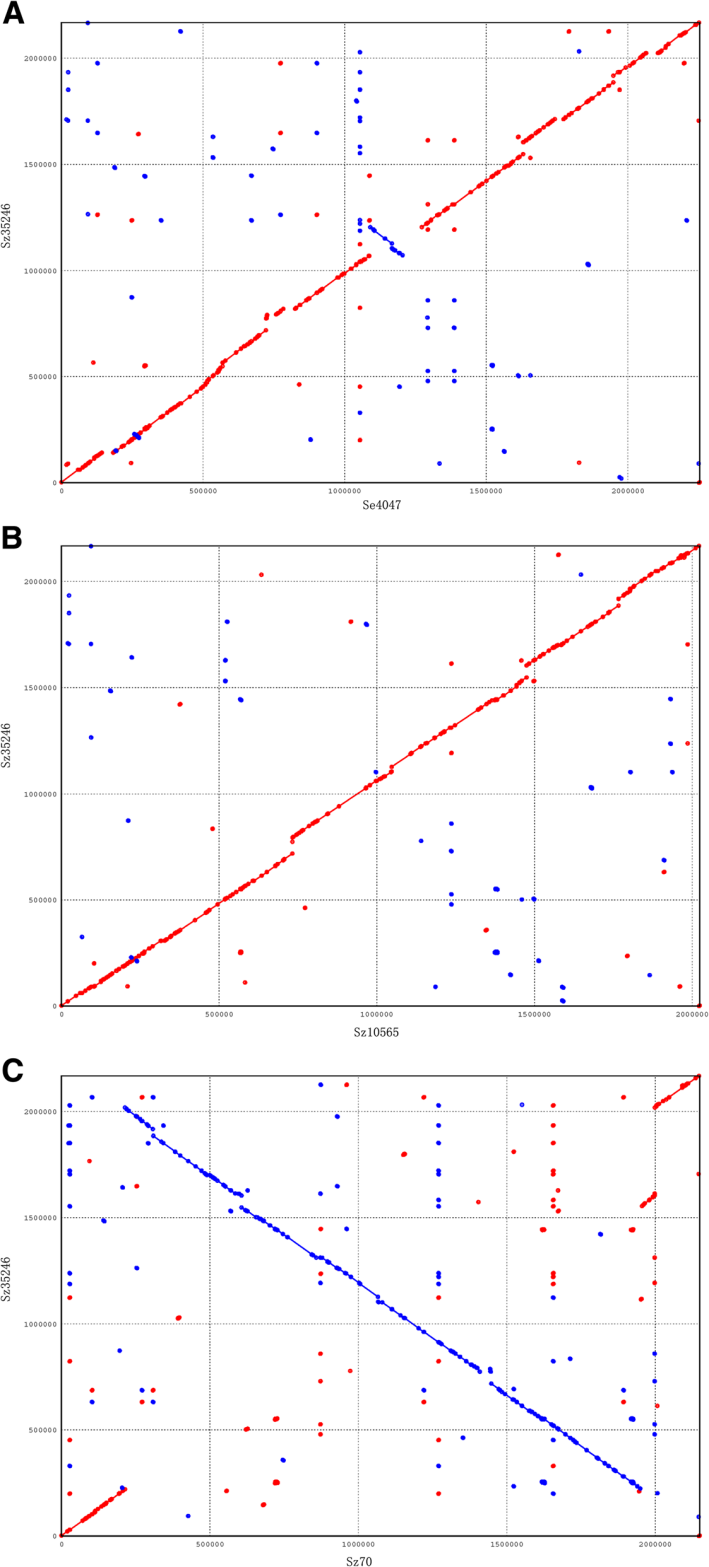
**Synteny between the Sz35246 genome and the Sz10565, Sz70 and Se4047 genomes, respectively.** The x-axis shows the position on Sz35246 genome; the y-axis shows the position on the Sz10565 genome (**A**), the position on the Sz70 genome (**B**) and the position on the Se4047 genome (**C**).

Comparative analysis of Sz35246 genome with three other genomes revealed that the evolution of Sz35246 has been driven by genomic rearrangements and HGT. X-alignment analysis of Sz35246 versus Sz10565 [12], Se4047 and Sz70 [13] revealed that small and large scale chromosome inversions have occurred during replication termination between Sz35246 and Se4047 and between Sz35246 and Sz70 (Figure 2). These genome rearrangements may influence the transcription of surrounding genes after the HGT process, which has contributed to the shaping of the Sz35246 genome.

**Figure 3:**
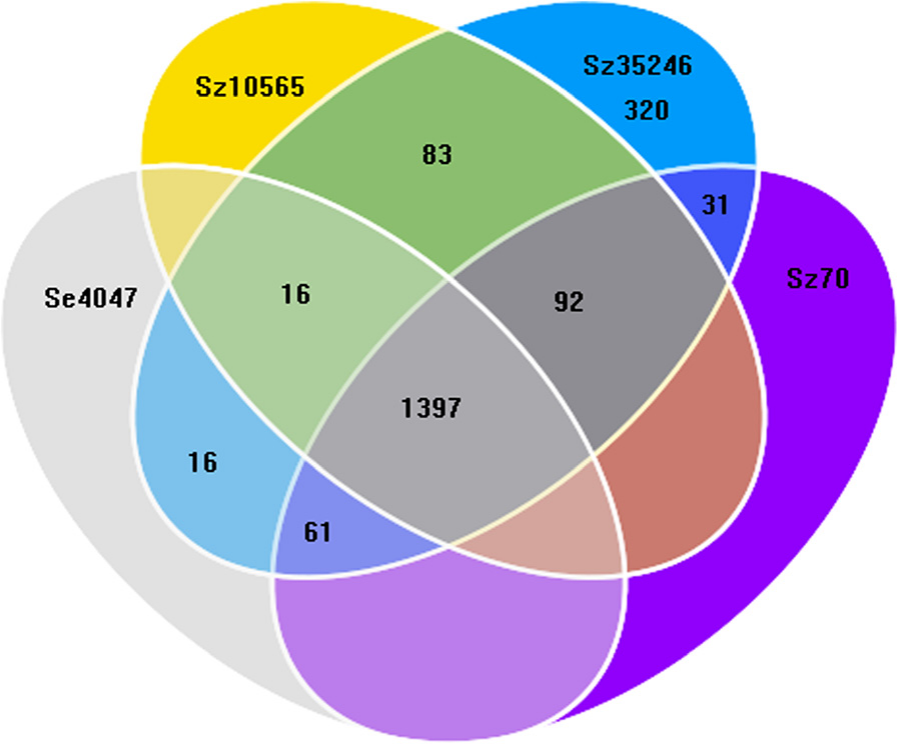
**The whole genome comparison among Sz35246 and Sz10565, Sz70, Se4047 genome.** Venn diagram representing unique and shared gene numbers among Sz35246, Sz10565, Sz70 and Se4047.

**Figure 4:**
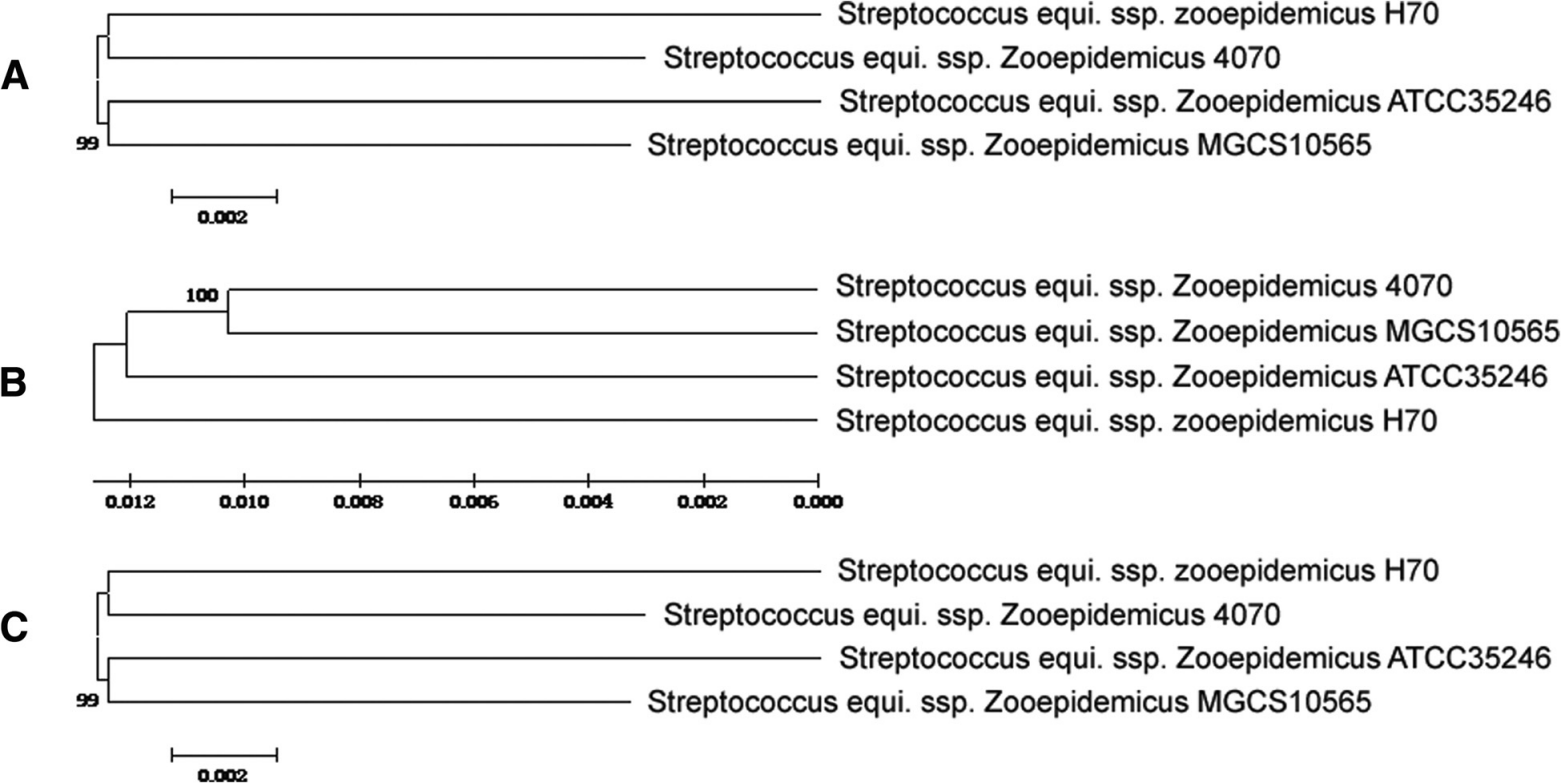
Phylogenetic trees were inferred using neighbour joining (A), maximum parsimony (B) and minimum evolution (C) among the four genomes based on concatenated alignments of 1397 orthologous proteins.

The comparative analysis of the Sz35246 genome with the three other genomes identified 1,397 orthologous genes that are shared by all four strains (Figure 3). In addition, 191, 184 and 93 genes are shared between Sz35246 and Sz10565, Sz70 and Se4047, respectively, suggesting that Sz35246 and Sz10565 are more closely related than the other strains. Furthermore, X-alignment analysis of Sz35246 versus Sz10565 [12], Se4047 and Sz70 [13] also suggested that Sz35246 is closer to Sz10565 than to the other two species. Phylogenetic trees of the four strains were constructed based on the sequences of the 1,397 orthologous genes using minimum evolution and neighbor joining phylogenic reconstruction methods available in the MEGA package (Figure 4). The phylogenic trees also indicated that Sz35246 is much closer to Sz10565 than to the other two species, which is consistent with the r genome-scale alignment analysis.

Further comparative analysis showed that 320 genes are specific to Sz35246, which include 197 (61%) that were annotated as “hypothetical protein”, among which 149 encode small proteins with lengths of no more than 100 amino acids (Additional file 3: Table S3). These small proteins are annotated as hypothetical proteins; however, certain highly conserved hypothetical proteins may play important roles in response to specific environmental stresses and host adaptation. For example, these small proteins have been reported to have evolved in response to specific environmental stress and to participate in the suppression of the type III secretion system [16]. The remaining functional genes encode 40 virulence proteins, 14 phage-associated proteins, eight transposases, five site-specific recombinases, a conjugation protein, a phage integrase, a phage recombinase, an IS transposase and a relaxase. These results suggest that the Sz35246 genome acquired these virulence genes through HGT, either by transduction with phages or by conjugation with plasmids or chromosomal fragments.

Furthermore, these Sz35246-specific genes are tightly clustered into four regions, varying in length from about 10 kb to 50 kb, which were as termed PAIs (SeseCisland_1~4) (Figure 1). An orthologous genes analysis between Sz35246 and Sz10565, Sz70, Se4047 confirmed these genomic islands are present in the Sz35246 genome only (Figure 1). The genes located in these four PAIs might be involved in Sz35246’s pathogenesis in causing swine streptococcosis and its strong virulence. These islands were further confirmed the annotation information and the co-linearity comparison of the Sz35246 genome with those of the three other genomes.

Significantly, sequence and annotation analyses of these islands revealed that SeseCisland_1, SeseCisland_2 and SeseCisland_3 contain the same type of virulence genes involved in the bacterial TA systems that have been reported to play subtle roles in the survival of bacteria under harsh natural environments [4,17]. Based on previous analyses of TA systems in *Escherichia coli* K12 [4], *Mycobacterium tuberculosis*[17] and *Mycobacterium smegmatis*[18]*,* we speculated that acquired-TA systems might play a positive role in survival of Sz35246 under different host environments. The RM system is used by bacteria to protect themselves from foreign DNA, such as bacteriophages and other viruses. Genes encoding RM system proteins, which include a restriction endonuclease and a restriction endonuclease control protein, were identified in a cluster in SeseCisland_4. Based on these results, we speculated that the acquired RM system might be involved in defense against infection by foreign DNA such as prophages and viruses. Thus, the PAIs may allow Sz35246 to adapt to various host stress conditions and to defend itself against infection by prophages, other bacteria and viruses. The expression and potential impact of these islands on the physiology, pathogenesis and host adaptation of Sz35246 are discussed below.

### I. SeseCisland_1: Phd/Doc TA system

**Figure 5:**
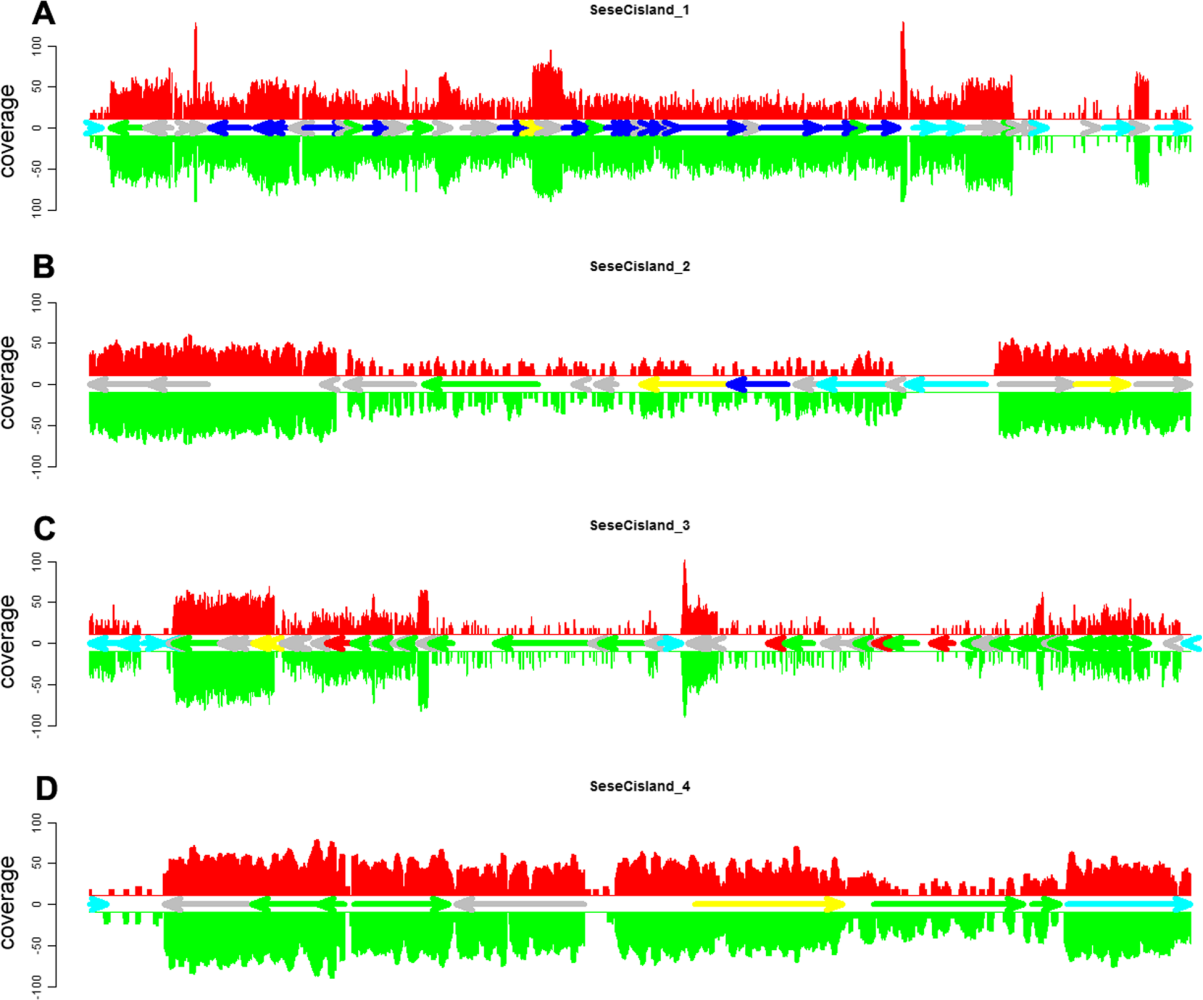
**Schematic representation of the putative PAIs and their expression in *S. zoopedemicus *ATCC35246 (A), SeseCisland_1; (B), SeseCisland_2; (C), SeseCisland_3; (D), SeseCisland_4.** The expression levels of in vitro and in vivo conditions are shown at single-nt resolution in red and green, respectively. All genes are color-coded based on the annotation information as follows: yellow, toxin-Antitoxin system (TA system); blue, phage associated protein; green, other virulence protein; red, virB4 components; cyan, mobile elements; grey, hypothetical protein.

SeseCisland_1 contains 54 genes (53,095 bp), 42 of which are Sz35246-specific genes, including mobile elements resembling the IS200 family transposase (SeseC_00874), a prophage site-specific recombinase resolvase family protein (SeseC_00919), a putative conjugal transfer protein (SeseC_00927), a conjugation protein (SeseC_00935) and a *tnpX* site-specific recombinase family protein (SeseC_00939), suggesting that this island is an integrative conjugative element (Additional file 4: Table S4 & Figure 5A). SeseCisland_1 contains 20 structural phage loci, indicating that MGEs, such as phages, are also implicated in HGT in *Streptococcus* species. Further analysis showed that the island has an abnormal GC skew and that the island-located genes have an average G+C content of 39.42% (Additional file 4: Table S4), which is significantly different from the mean value for the genome (41.65%) (p=0.002).

**Figure 6:**
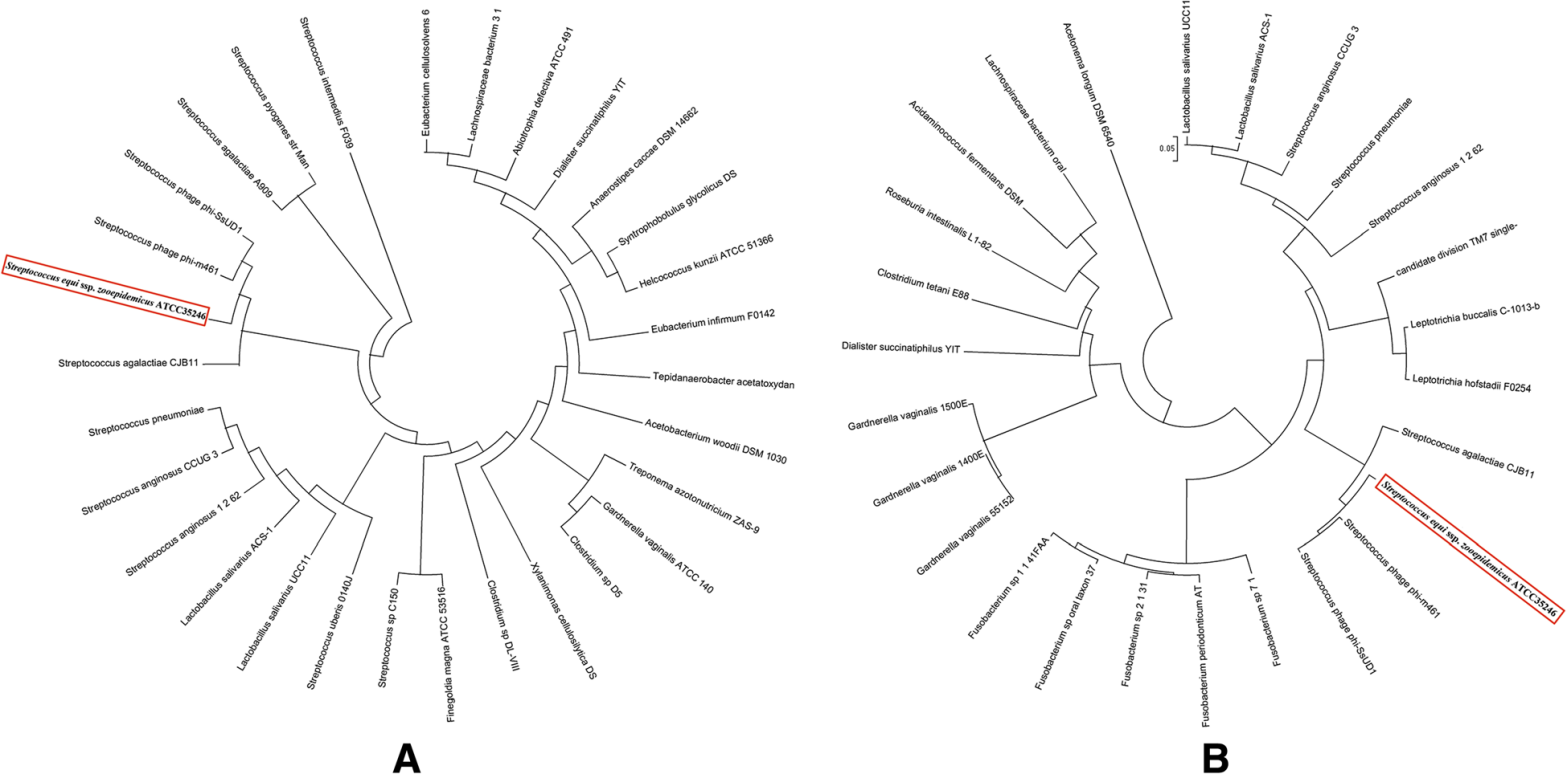
**Phylogeny of antitoxin Phd and addiction module killer Doc protein sequences.** Thirty phd toxin sequences (**A**) and twenty-six anti-toxin sequences (**B**) from loci of bacterial chromosomes, plasmids and phages were aligned. The sequences were aligned with clustalW and the genetic relationships inferred using the unweighted pair group method with arithmetic mean (UPGMA) implanted in MEGA 4.0 software.

A major feature within SeseCisland_1 is the presence of two genes (SeseC_00898 and SeseC_00899) encoding proteins homologous to addiction module antitoxin Phd protein and killer Doc protein, respectively. The Phd-Doc TA system is the Type II TA system that first identified in bacteriophage P1 [19]. Phylogenetic trees based on Phd/Doc proteins also suggested that Phd/Doc proteins are highly homologous to those of Streptococcus phage phi-m461 and phi-SsuD1 (Figure 6). The Phd/Doc mRNAs are co-expressed from the same promoter and both are translated into proteins. These proteins form a stable TA complex to block the functions of the Doc toxin. Doc is a toxin that kills plasmid-free segregants, and Phd is an unstable antidote that neutralizes the toxin. Doc inhibits translation elongation by association with the 30S ribosomal subunit [20]. Under stress conditions or host change, Phd is degraded by ATP-dependent serine proteases, such as ClpXP protease [21], resulting in freeing of the toxin from the TA system and inducing cell growth inhibition and cell death [22,23]. Interestingly, a gene (SeseC_00903) encoding a protein homologous to the E*. coli* Clpxp protein is present in this island. Doc toxins are expressed in two conditions (in vivo and in vitro) (Figure 5A), which agrees with previous reports that Doc causes cell growth and death by inhibiting translation without affecting transcription and replication. The observations and results reported here support the hypothesis that SeseCisland_1 helps Sz35246 to adapt to environmental and host changes.

### II. SeseCisland_2: Fic/Doc TA system

**Figure 7:**
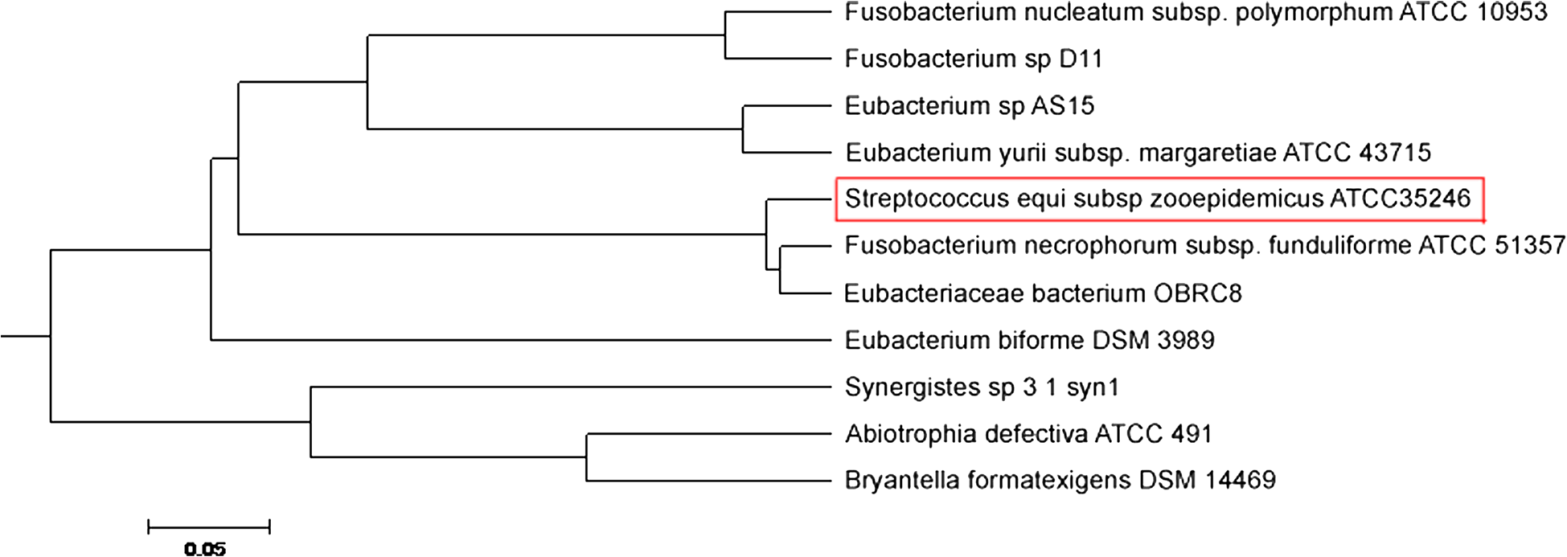
**Phylogeny of Fic/DOC domain protein sequences.** Twelve protein sequences of bacteria were aligned using clustalW and the genetic relationship trees were constructed with MEGA4.0 software as well as Figure 6.

SeseCisland_2 contains an important open reading frame (ORF), SeseC_01334, which encodes a protein with a filamentation induced by cAMP (Fic) domain (Additional file 5: Table S5 and Figure 5B). The Fic domain is classified together with a second family of sequences, doc (death on curing), in the Pfam protein families database [24]. The Fic/Doc family protein sequences are aligned against this protein present inside other bacteria. Interestingly, phylogenetic analysis revealed that the Fic/Doc protein is homologous to that of *Fusobacterium nucleatum subsp fusiforme* (Figure 7). Fic/Doc family proteins are known as members of a TA system, the functional sites are common to both families [25]. The Fic protein has been reported to be involved in cell division and synthesis of folate, indicating that the Fic protein and cAMP are involved in a regulatory mechanism of cell division via folate metabolism [26,27]. Fic family virulence proteins may be important in many bacterial pathogens. For example, the immunoglobulin-binding protein A (IbpA) of *Histophilus somni* contains a direct repeat of two Fic domains, and mutation of IbpA or just the fic domain of IbpA decreased the virulence of this bacteria. The Fic domain has been shown to covalently modify host Rho GTPases with AMP, which may explain how the Fic domain influences bacterial virulence [28]. Thus, the Fic family protein in SeseCisland_2 may be involved in the pathogenicity of Sz35246.

SeseCisland_2 encodes 16 genes (17,293 bp), 10 of which are Sz35246-specific. SeseCisland_2 also contains certain mobile elements, including an endonuclease relaxase (SeseC_01323), a bacterial mobilization protein (SeseC_01324) integrase/recombinase (SeseC_01328) and transposase protein (SeseC_01332). Thus, we speculate that this region also plays important roles in bacterial adaptation, virulence and physiology.

### III. SeseCisland_3: ϵ/ζ TA system

SeseCisland_3 contains 21 Sz35246-specific genes (Additional file 6: Table S6 and Figure 5C), the most notable of which are two genes annotated as Type II TA system genes encoding a ζ toxin protein (SeseC_01875) and an ϵ antitoxin protein (SeseC_01876). VirB4/VirB6/VirD4 components (SeseC_01908, SeseC_01912 SeseC_01914 and SeseC_01916) from the type IV secretion system (T4SS) are also present. Additionally, virulence-associated factors, such as glucan-binding protein and abortive infection protein, are also encoded by this region. All the virulence genes are expressed under in vitro and in vivo conditions. Several MGEs such as site-specific recombinases (SeseC_01863, SeseC_01864 and SeseC_01865) and transposases (SeseC_01867&SeseC_001869) were also identified in this island. The bioinformatics analysis showed that a Type II TA system, a type IV secretion system and other virulence genes were present in this island, which may contribute directly to the bacterium’s pathogenicity and host adaption.

ϵ/ζ systems ensure stable plasmid inheritance by inducing death in plasmid-deprived offspring cells. Members of the ϵ/ζ systems have been found on resistance plasmids in major human pathogens [29,30]. By contrast, chromosomally encoded ϵ/ζ systems were reported to contribute to virulence of pathogenic bacteria. Brown *et al*. compared clinical serotype 3 isolates with ζ toxin gene knockout strains in mixed systemic and respiratory infections of mice, and thus connected the ζ toxin with virulence in *Streptococcus pneumonia*[31]. The ϵ/ζ system also exists in the 89 k pathogenicity island of *Streptococcus suis* serotype 2. This bacterium is an important zoonotic pathogen, causing more than 200 cases of severe human infection worldwide [32]. The ζ toxin is inhibited by its cognate antitoxin, ϵ. The structure of the complex of ζ toxin inactivated by ϵ antitoxin (ϵ_2_ζ_2_) was solved by X-ray crystallography [33]. Upon degradation of ϵ, the ζ toxin is released, allowing this enzyme to inhibit bacterial cell wall synthesis, which eventually triggers autolysis [34]. The toxic effect of the ζ toxin has also been demonstrated in a diverse array of organisms, including *Saccharomyces cerevisiae*[35].

**Figure 8:**
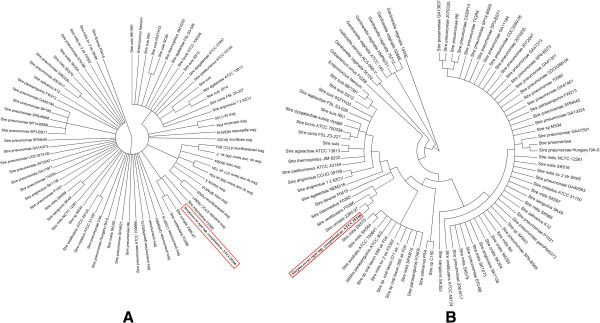
**Phylogeny of zeta toxin (PezT) (A) and epsilon antitoxin (PezA) (B) protein sequences.** Eighty-eight zeta toxin protein sequences (**A**) and seventy-two epsilon antitoxin protein sequences (**B**) from *Streptococcus*, *Enterococcus*, *Oribacterium*, *Veillonella* and *Gardnerella* bacterial species were aligned. The genetic relationships were determined as detailed in Figure 6.

Phylogenetic analysis of ζ proteins and ϵ antitoxin proteins showed that the proteins from Sz35246 are highly homologous to those of *Streptococcus urinalis* 2285*–*97, *Streptococcus intermedius* F0395 and *Streptococcus vestibularis* F0396 and widely distributed in many bacteria (Figure 8). This broad distribution has been reported that the zeta toxin family on plasmids [21,36,37], bacterial chromosomes [23,38] and in *Streptococcus pneumonia* and *Streptococcus suis* serotype 2 PAIs. The broad distribution of this system within the bacterial kingdom suggests that it uses a ubiquitous bacteriotoxic mechanism to overcome host defenses and environmental changes. On the other hand, we hypothesized that horizontal transfer of this island may occur through T4SS-mediated conjugation process, because four genes products display similarities to Streptococcus T4SS components.

### IV SeseCisland_4: RM system and virulence island

SeseCisland_4 contains eight Sz35246-specific genes (from a total of 10 genes), the two mobile elements (SeseC_02358, SeseC_02362) are transposase IS1167 and phage integrase (Additional file 7: Table S7 and Figure 5D), suggesting that this island has been acquired by HGT from another microorganism. The major feature of this island is three strain specific genes (SeseC_02360, SeseC_02361 and SeseC_02362) that were annotated as RM system proteins, which protect bacteria from foreign DNA, such as bacteriophages. The RM system is strategy that permits bacteria to live in difference environments [39], allowing bacteria erect a barrier to gene transfer and making them resistant to phage infection [40]. Taken together, these data suggest that the RM systems is a remarkable characteristic of Sz35246 and is probably involved in the adaptation of these bacteria to different environmental conditions.

### Relationship between PAIs and Sz35246 virulence

**Figure 9:**
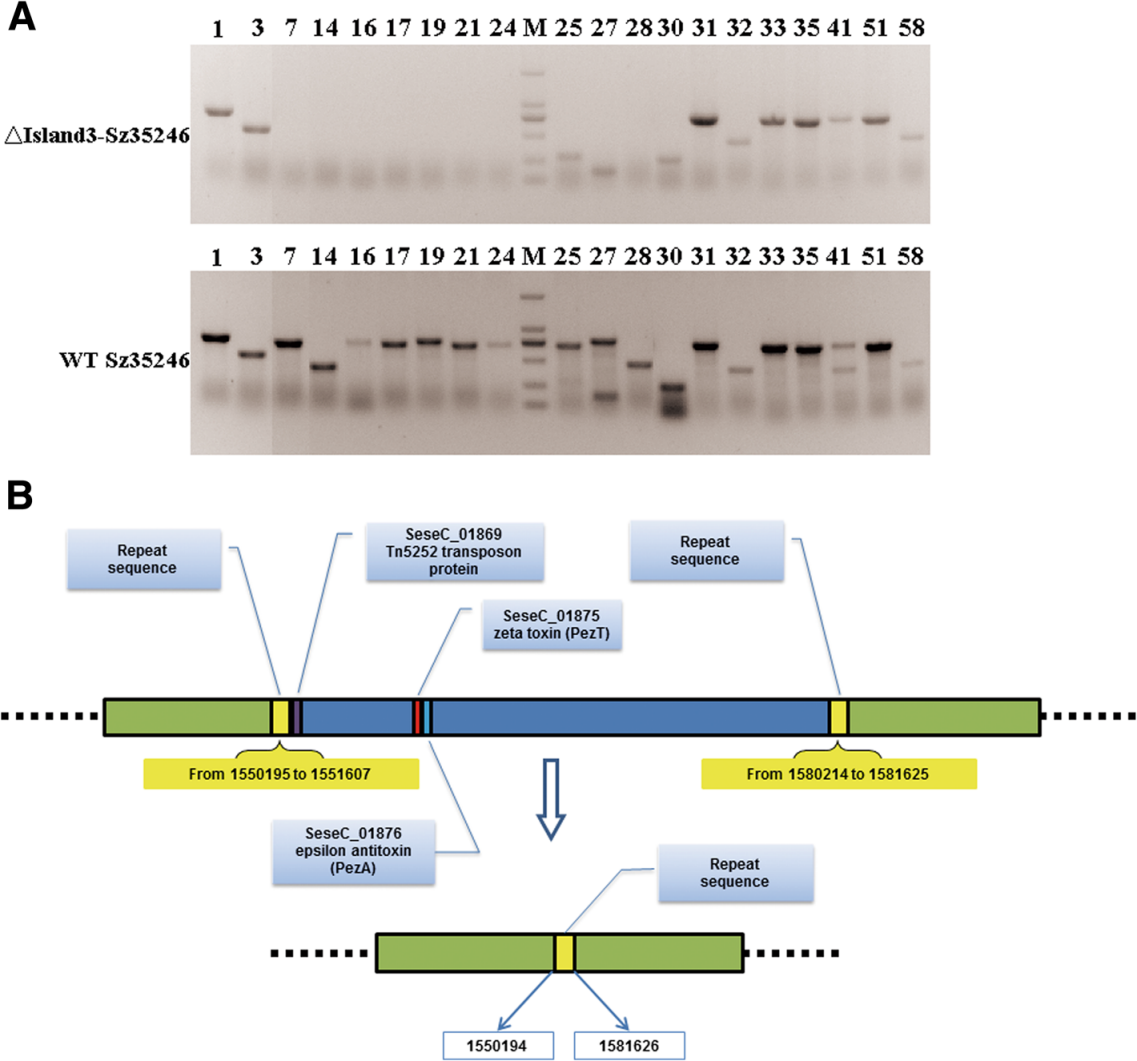
**PCR detection and schematic diagram of partial SeseCisland_3 deletion.** (**A**) PCR detection of genes located in SeseCisland_3 deleted region (markers are 2000 bp, 1000 bp, 750 bp, 500 bp, 250 bp and 100 bp), the number of genes corresponds to Additional file 8: Table S8. (**B**) Genomic organization of the partial SeseCisland_3 deletion locus and its flanking repeat sequences in Sz35246. The fragment from 1551608 to 1580213 was knocked out, including the zeta toxin (PezT) and epsilon antitoxin (PezA) genes. The deleted region started with a Tn5252 transposon protein gene and is flanked by two repeated regions. After the reciprocal recombination, only one repeat region remained.

**Figure 10:**
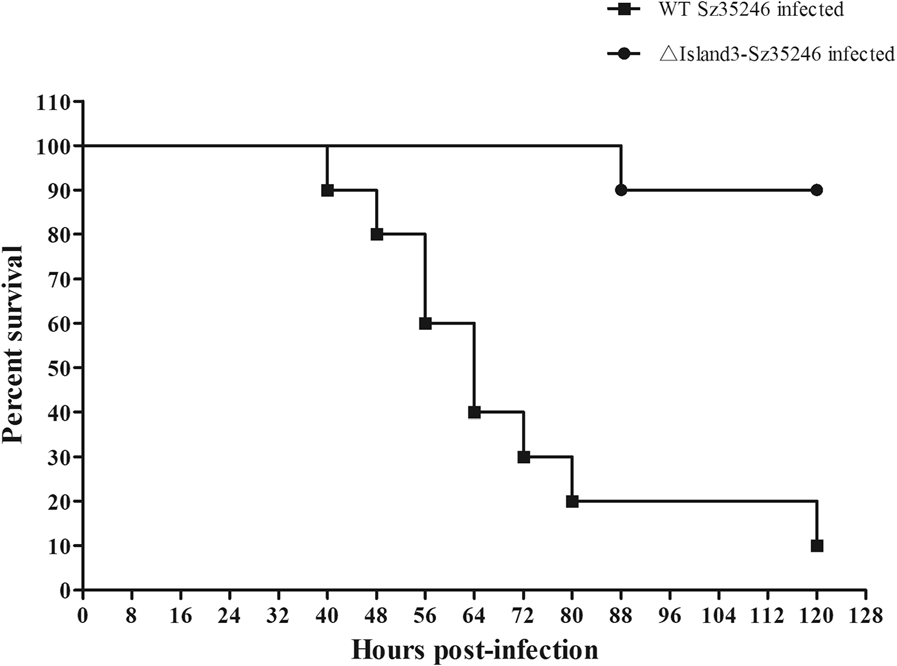
**Survival curves for ICR mice infected with the wild-type Sz35246 and ∆Island3-Sz35246.** Two groups of eight-week-old ICR mice were inoculated i.p. with 2.5×10^5^ CFU bacteria, and mouse survival was monitored over a 5-day period. Data are expressed as the mean percentage of live animals in each group (n = 10). The virulences of these two strains were significantly different (P<0.05).

To prove that the genes located within the PAIs affect the virulence of Sz35246, we deleted part of SeseCisland_3 from SeseC_01869 to SeseC_01898. PCR was used to confirm the deletion (Figure 9A and Additional file 8: Table S8), sequencing results showed that exactly 28,606 bp of SeseCisland_3 was deleted, including the genes belong to the ϵ /ζ TA system (Figure 9B). The deleted region started with Tn5252 transposon gene (SeseC_01869), and two repeat sequences, including transposase genes (SeseC_01867 and SeseC_01901), were located at the flank of the deleted region. These two repeat sequences and the Tn5252 transposon gene formed the structural basis for deleting such a long fragment. The mutant strain ∆Island3-Sz35246 and wild-type Sz35246, were used to infect ICR mice to evaluate the influence of partial PAI deletion on bacterial virulence. The percent survival significantly increased in ∆Island3-Sz35246 infected mice (Figure 10), 5 days post-infection, only one of the ten mice was dead; however, of the mice infected with wild-type Sz35246, only one was alive. The survival curve indicated that partial deletion of a PAI did affect the virulence of Sz35246, and that some of these genes in the PAI are important for bacterial pathogenicity. Genes located in the other three PAIs require further study to determine their role in bacterial virulence.

### Other potential virulence genes dispersed in the Sz35246 genome

Strain Sz70 was isolated from a nasal swab taken from a healthy thoroughbred racehorse in Newmarket, England, in 2000 [41]. A genome wide comparison of Sz35246 with Sz70 identified Sz35246-specific genes, some of which may be involved in the virulence of Sz35246 and may provide clues as to why Sz35246 causes such a serious swine streptococcosis but other strains do not. Virulence-associated protein E (vapE, SeseC_01325), which was originally identified in *Dichelobacter nodosus*[42], is part of a vap region of *D. nodosus* that is associated with virulence [43]. The mechanism by which VapE affects virulence has not been determined yet, but the presence of an integrase gene XerC (SeseC_01328) immediately upstream of vapE, suggested a role for bacteriophages in the evolution and transfer of these bacterial virulence determinants; i.e., it is possible that exchange of this putative virulence factor with other bacteriophages could take place [44]. Moreover, a vapE-like gene has also been identified in a pathogenicity island of *Staphylococcus aureus*[45]*.* The pathogens of a footrot outbreak in a Debre Zeit swine farm were identified as *Staphylococcus aureus* and *Dichelobacter nodosus*, both bacteria contain the vapE gene. VapE has not been identified in other strains of *S. zooepidemicus,* but only in Sz35246. This gene may be related to Sz35246 pathogenicity towards pig. The role of the vapE gene in the virulence of Sz35246 remains to be clarified.

Adherence is an essential requirement for invasion of cells by bacterial pathogens. Long extracellular structures resembling fimbriae mediate adhesion to components of the host extracellular matrix, such as collagen and fibronectin. We identified seven Sz35247 unique proteins that contain an LPXTG motif (found in cell wall anchor domains), including collagen-like protein SclZ.1 (SeseC_00092), fibrinogen- and Ig-binding protein precursor (SeseC_00180), cell surface protein (SeseC_00619), T-antigen-like fimbrial structural subunit protein (SeseC_02472), putative cell surface protein (SeseC_02304), InlA-like domain containing cell surfaced-anchored protein (SeseC_01462) and collagen-like surface-anchored protein SclE (SeseC_00246). All of these proteins are anchored on the bacterial surface and may be involved in bacterial adhesion and invasion.

Fibronectin (Fn)-binding proteins have been reported to mediate the invasion of host cells without the need for other bacterial factors [46]. Fn, which has received much attention as a target of bacterial adhesins, it is a glycoprotein found in the extracellular matrix and body fluids of vertebrates. Fn-binding proteins are found in *Streptococcus pyogenes* (SfbI/F1), *Staphylococcus aureus* (FnBPA and FnBPB), *Streptococcus dysgalactiae* (FnBA and FnBB), and other bacterial species [47]. In previous research, an *fnz* gene was found in *S. zooepidemicus* and a *sfs* gene was only found in *S. equi,* both of which genes encode a cell surface protein that binds Fn [48]. The *sfs* gene (SeseC_00464) was identified in Sz35246 for the first time. The transcriptome data showed that the *sfs* gene was upregulated infection of a host by Sz35246 (in vivo). Presumably, the expression of this gene promoted bacterial pathogenicity by inhibiting the binding between collagen and Fn.

The Sz35246 and Sz10565 genomes both have the Fim III operon (type II fimbriae) (SeseC_02471-02473 and SeseC_02475). The structural proteins of type III fimbriae have an amino-terminal secretion signal and a carboxy-terminal sorting signal, and their assembly into fimbriae is dependent on the adjacently encoded dedicated sortases [49]. Sz70 contains two loci that encode genes putatively required for pilus expression, but lacks this putative pilus locus. Recent studies of *Salmonella enterica* revealed that the presence of fimbriae increases the ability of host-restricted bacteria to invade normally restrictive cells [50]. Thus, we hypothesize that the presence of the Fim III operon might be associated with breaking host-restriction by *S. zooepidemicus*.

## Conclusions

The genome and expression analysis of Sz35246 provided fundamental information on the physiology and potential pathogenic capacity of this bacterium. The comparison of the genomes of Sz35246, Se4047, Sz10565 and Sz70 identified gens that are specific to Sz35246. These genes may be related to the bacterium’s pathogenic function, including causing swine streptococcosis and breaking host-restriction. We identified novel MGEs, which may have been involved in the evolution of Sz35246. The presence of the elements and the phylogenetic analysis indicated that this genome has been shaped by chromosomal inversion, recombination and HGT events. Sz35246 probably acquired its PAIs and certain specific genes through HGT. The presence of TA systems exists in three of genomic islands of Sz35246 may be related to this strains pathogenicity. Study of these systems will form the basis of our future research. The availability of the complete Sz35246 genome sequence will facilitate further studies of this pathogen and the development of diagnostics and vaccines.

## Methods

### Strain and growth conditions

*S. zooepidemicus* ATCC35246 was isolated from a dead pig in Sichuan, China.. To prepare total cellular DNA from *S. zooepidemicus* ATCC35246, bacteria were grown in Bacto^™^ Todd-Hewitt Broth at 37°C, in a 10% CO_2_ atmosphere. Total cellular DNA was isolated from the mid-exponential (OD_600_= 0.6) phase culture using a Genomic Purification System (Promega).

### Preparation of RNA for transcriptome analysis

#### From pure culture

cultures for preparing RNA samples were grown overnight at 37°C under aerobic conditions in liquid medium with shaking. Overnight pre-cultures were diluted in liquid medium and incubated at 37°C under aerobic conditions with shaking. Exponentially growing cells (OD_600_= 0.6) were harvested by centrifugation for 10 min at 10,000 rpm at 4°C. Total RNA was extracted as previously described [51]. RNA quality was assessed by determining the OD260/280 ratio with a Nanodrop 2000 (Thermo) and by visualization following agarose gel electrophoresis.

#### From infected mice organs

specific pathogen-free female BALB/c mice were intravenously infected with *S. zooepidemicus* ATCC35246 [52]. At 24 h post-infection, the mice were sacrificed and dissected. The livers and spleens were harvested and immediately frozen in liquid nitrogen. The organs were stored at −80°C. Before RNA isolation, the organs were thawed on ice and homogenized in 20 ml of an ice-cold solution composed of 0.2 M sucrose/0.01% SDS. The homogenate was gently centrifuged for 20 min at 300 rpm and filtered to remove large tissue debris. The tissue suspension was centrifuged for 20 min at 8000 rpm to pellet the bacteria. Centrifugations were performed at 4°C. Bacterial RNA extraction was performed as previously described [51]. RNA quality was assessed by determining the OD260/280 ratio with a Nanodrop 2000 (Thermo) and by visualization following agarose gel electrophoresis.

### Genome sequencing and annotation

Whole-genome sequencing was performed with the Roche 454 genome sequencer FLX system and assembled with Newbler (version 2.0.01.14). The detailed sequencing and assembly methods have been described previously [52]. The complete genome sequence of *S. zooepidemicus* strain ATCC35246 has been deposited in the GenBank database with the accession number CP00290. The replication origin (*oriC*) was identified with Ori-Finder software [53]. Protein-coding genes were predicted with Glimmer 3.02 [54] using the default settings and a cutoff at 90 nt. Annotation of these genes was performed by homology searches in the NCBI nonredundant protein database with 80% overlap (E_value<1e-10), in the cluster of orthologous groups (COG) database [55], the InterPro member (InterProScan) databases [56] and the Kyoto encyclopedia of genes and genomes (KEGG) pathway database [57], respectively. The tRNA genes and rRNA genes were identified using the tRNAScan-SE tool [58], and RNAmmer 1.2 [59], respectively. Finally, genome annotation and the structure of the predicted genes were manually refined.

### Comparative genomic analysis

Sequences and protein coding sequences for each strain (MGCS10565: CP001129.1; 4047:FM204883.1; H70:FM204884.1) were retrieved from NCBI (http://www.ncbi.nlm.nih.gov). The genomic co-linearity of four genome sequences was generated using the MUMmer 3 package [60]. Orthologous proteins were identified with Inparanoid and MultiParanoid [61]. The CLUSTAL W software [62] and MEGA4 software [63] were used to align the concatenated sequences from all orthologs and to construct phylogenetic trees. The Artemis Comparison Tool (ACT) [64] was used to view the overall comparison of *S. zooepidemicus* strain ATCC35246 and *S. zoopidemicus* MGCS10565, *S. zooepidemicus* H70 and *S. equi* 4047.

### SOLiD RNA-seq library construction, sequencing and gene expression analysis

The standard protocol from SOLiDTM Small RNA Expression Kit (ABI) was used to construct the RNA-Seq library and sequencing was performed on an ABI SOLiD 4.0 sequencer. Reads with a quality value greater than 8 were mapped to the *S. zooepidemicus* strain ATCC35246 genome using the SOLiD™ System Analysis Pipeline Tool ,allowing mismatches up to five bases. The detailed mapping methods have been described previously [63] and rRNA reads were filtered before mapping. The expression level of genes was calculated by read counts normalized with the total mapped reads and the gene length was calculated using the RPKM method [65]. The differential expressions of genes between the in vitro and in vivo libraries were analyzed based on the DEGseq modeling methods [66].

### Identification of pathogenicity islands (PAIs)

The PAIs of *S. zooepidemicus* strain ATCC35246 were identified according to the following criteria: First, GC content and GC skew were determined and regions showing differences from the average of the whole genome indicated potential PAI loci. Second, the PAI locus was present in ATCC35246, but was absent or scattered in the other three species. Third, mobility genes, such as integrases, transposases, IS elements were present at the boundaries of the locus. Four, virulence genes were located in the locus. Finally, these loci were confirmed using IslandViewer, an genomic island predictor that integrates three methods: IslandPick, IslandPath-DIMOB, and SIGI-HMM [67].

### Construction of partial SeseCisland_3 knockout strain, ∆Island3-Sz35246

To construct **∆**pSET4s-LR plasmid, the upstream (LA) and downstream (RA) fragments of the Sz35246 target region were amplified. These two fragments were linked by fusion PCR and inserted into the pSET4s plasmid. Competent Sz35246 cells were subjected to electrotransformation with **∆**pSET4s-LR plasmid and positively transformed cells were selected at 28°C in the presence of spectinomycin. Bacteria at the mid logarithmic growth phase were diluted with THB containing spectinomycin and cultured at 28°C to the early logarithmic phase. The culture was then shifted to 37°C and incubated for 4 h. Subsequently, the cells were spread on THB and incubated at 28°C. Temperature resistant colonies were screened at 37°C for the loss of vector-mediated spectinomycin resistance. The putative double crossover homologous recombinant mutants and some of the deleted genes in SeseCisland_3 were detected by PCR.

### In vivo challenges of ICR mice

The Laboratory Animal Monitoring Committee of Jiangsu Province approved the experimental protocols. Two groups of eight-week-old ICR mice (10 animals per group) were used for in vivo infection studies. The wild-type Sz35246 and **∆**Island3-Sz35246 were cultured with THB medium (Difco) at 37°C, with shaking at 180 rpm, separately. When the OD_600_ reached 0.6, bacteria were pelleted, resuspended in PBS and diluted appropriately to 1.25 × 10^6^ CFU/ml (5×LD_50_ per 0.2 ml, LD_50_=5×10^4^ CFU/ml) [68]. Mice were injected with 0.2 ml of liquid bacterial suspension. Survival was monitored for 5 days. Survival curves and statistical analysis were made by GraphPad Prism (Version 5.02).

## Competing interests

The authors declare that they have no competing interests.

## Authors’ contributions

ZM and JG carried out the genome sequencing, participated in the virulence genes studies, sequence alignment, transcriptome analysis and drafted the manuscript. LY and YL prepared the bacterial DNA and RNA. ZM and BX carried out the gene knockout and animal experiments. RJ and QM participated in the analysis of PAIs. HF and SH conceived of the study, and participated in its design and coordination and helped to draft the manuscript. All authors read and approved the final manuscript.

## Supplementary Material

Additional file 1: Table S1 Genome information for *S. zoopedemicus* ATCC35246. Click here for file

Additional file 2: Table S2 Comparative gene expression analysis in vitro and in vivo for *S. zoopedemicus* ATCC35246. Click here for file

Additional file 3: Table S3 *S. zoopedemicus* ATCC35246-specific genes. Click here for file

Additional file 4: Table S4 Genes in SeseCisland_1 of the *S. zoopedemicus* ATCC35246 genome. Click here for file

Additional file 5: Table S5 Genes in SeseCisland_2 of the *S. zoopedemicus* ATCC35246 genome. Click here for file

Additional file 6: Table S6 Genes in SeseCisland_3 of *S. zoopedemicus* ATCC35246 genome. Click here for file

Additional file 7: Table S7 Genes in SeseCisland_4 of the *S. zoopedemicus* ATCC35246 genome. Click here for file

Additional file 8: Table S8 PCR detection of genes located in SeseCisland_3 deleted region. Red: Genes still existed in Sz35246 SeseCisland_3. Yellow: Knocked out genes of Sz35246 SeseCisland_3. Click here for file

## References

[B1] Deng W Puente JL Gruenheid S Li Y Vallance BA Vazquez A Barba J Ibarra JA O'Donnell P Metalnikov P Dissecting virulence: systematic and functional analyses of a pathogenicity island Proc Natl Acad Sci USA 2004 101 10 3597 3602 10.1073/pnas.0400326101 14988506 PMC373508

[B2] Li M Shen X Yan J Han H Zheng B Liu D Cheng H Zhao Y Rao X Wang C GI-type T4SS-mediated horizontal transfer of the 89K pathogenicity island in epidemic *Streptococcus suis* serotype 2 Mol Microbiol 2011 79 6 1670 1683 10.1111/j.1365-2958.2011.07553.x 21244532 PMC3132442

[B3] Mutschler H Meinhart A epsilon/zeta systems: their role in resistance, virulence, and their potential for antibiotic development J Mol Med (Berl) 2011 89 12 1183 1194 10.1007/s00109-011-0797-4 21822621 PMC3218275

[B4] Yamaguchi Y Park JH Inouye M Toxin-antitoxin systems in bacteria and archaea Annu Rev Genet 2011 45 61 79 10.1146/annurev-genet-110410-132412 22060041

[B5] Brzozowska I Brzozowska K Zielenkiewicz U Functioning of the TA cassette of streptococcal plasmid pSM19035 in various Gram-positive bacteria Plasmid 2012 68 1 51 60 10.1016/j.plasmid.2012.01.010 22309878

[B6] Timoney JF The pathogenic equine streptococci Vet Res 2004 35 4 397 409 10.1051/vetres:2004025 15236673

[B7] Abbott Y Acke E Khan S Muldoon EG Markey BK Pinilla M Leonard FC Steward K Waller A Zoonotic transmission of *Streptococcus equi* subsp. *zooepidemicus* from a dog to a handler J Med Microbiol 2010 59 Pt 1 120 123 19745031 10.1099/jmm.0.012930-0

[B8] Eyre DW Kenkre JS Bowler IC McBride SJ *Streptococcus equi* subspecies *zooepidemicus* meningitis--a case report and review of the literature Eur J Clin Microbiol Infect Dis 2010 29 12 1459 1463 10.1007/s10096-010-1037-5 20820836

[B9] Feng ZG Hu JS Outbreak of swine streptococcosis in Sichan province and identification of pathogen Anim Husbandry Vet Med Lett 1977 2 7 12

[B10] Liu PH Shen FS Wang YK Zhang SH Identification of swine Streptococcus isolates in Shanghai Chin J Vet Med 2001 21 42 46

[B11] Ma Z Geng J Zhang H Yu H Yi L Lei M Lu CP Fan HJ Hu S Complete genome sequence of *Streptococcus equi* subsp. *zooepidemicus* strain ATCC 35246 J Bacteriol 2011 193 19 5583 5584 10.1128/JB.05700-11 21914890 PMC3187426

[B12] Beres SB Sesso R Pinto SW Hoe NP Porcella SF Deleo FR Musser JM Genome sequence of a Lancefield group C *Streptococcus zooepidemicus* strain causing epidemic nephritis: new information about an old disease PLoS One 2008 3 8 e3026 10.1371/journal.pone.0003026 18716664 PMC2516327

[B13] Holden MT Heather Z Paillot R Steward KF Webb K Ainslie F Jourdan T Bason NC Holroyd NE Mungall K Genomic evidence for the evolution of *Streptococcus equi*: host restriction, increased virulence, and genetic exchange with human pathogens PLoS Pathog 2009 5 3 e1000346 10.1371/journal.ppat.1000346 19325880 PMC2654543

[B14] Shelburne SA 3rd Sumby P Sitkiewicz I Okorafor N Granville C Patel P Voyich J Hull R DeLeo FR Musser JM Maltodextrin utilization plays a key role in the ability of group A Streptococcus to colonize the oropharynx Infect Immun 2006 74 8 4605 4614 10.1128/IAI.00477-06 16861648 PMC1539623

[B15] Giammarinaro P Paton JC Role of RegM, a homologue of the catabolite repressor protein CcpA, in the virulence of *Streptococcus pneumoniae* Infect Immun 2002 70 10 5454 5461 10.1128/IAI.70.10.5454-5461.2002 12228270 PMC128313

[B16] Wang F Xiao J Pan L Yang M Zhang G Jin S Yu J A systematic survey of mini-proteins in bacteria and archaea PLoS One 2008 3 12 e4027 10.1371/journal.pone.0004027 19107199 PMC2602986

[B17] Wayne LG Dormancy of *Mycobacterium tuberculosis* and latency of disease Eur J Clin Microbiol Infect Dis 1994 13 11 908 914 10.1007/BF02111491 7698116

[B18] Pandey DP Gerdes K Toxin-antitoxin loci are highly abundant in free-living but lost from host-associated prokaryotes Nucleic Acids Res 2005 33 3 966 976 10.1093/nar/gki201 15718296 PMC549392

[B19] Jensen RB Gerdes K Programmed cell death in bacteria: proteic plasmid stabilization systems Mol Microbiol 1995 17 2 205 210 10.1111/j.1365-2958.1995.mmi_17020205.x 7494469

[B20] Liu M Zhang Y Inouye M Woychik NA Bacterial addiction module toxin Doc inhibits translation elongation through its association with the 30S ribosomal subunit Proc Natl Acad Sci USA 2008 105 15 5885 5890 10.1073/pnas.0711949105 18398006 PMC2311363

[B21] Bolhuis H Palm P Wende A Falb M Rampp M Rodriguez-Valera F Pfeiffer F Oesterhelt D The genome of the square archaeon Haloquadratum walsbyi : life at the limits of water activity BMC Genomics 2006 7 169 10.1186/1471-2164-7-169 16820047 PMC1544339

[B22] Aizenman E Engelberg-Kulka H Glaser G An *Escherichia coli* chromosomal "addiction module" regulated by guanosine [corrected] 3',5'-bispyrophosphate: a model for programmed bacterial cell death Proc Natl Acad Sci USA 1996 93 12 6059 6063 10.1073/pnas.93.12.6059 8650219 PMC39188

[B23] Christensen SK Maenhaut-Michel G Mine N Gottesman S Gerdes K Van Melderen L Overproduction of the Lon protease triggers inhibition of translation in *Escherichia coli*: involvement of the yefM-yoeB toxin-antitoxin system Mol Microbiol 2004 51 6 1705 1717 10.1046/j.1365-2958.2003.03941.x 15009896

[B24] Finn RD Tate J Mistry J Coggill PC Sammut SJ Hotz HR Ceric G Forslund K Eddy SR Sonnhammer EL The Pfam protein families database Nucleic Acids Res 2008 36 D281 288 Database issue 10.1093/nar/gkn226 18039703 PMC2238907

[B25] Garcia-Pino A Christensen-Dalsgaard M Wyns L Yarmolinsky M Magnuson RD Gerdes K Loris R Doc of prophage P1 is inhibited by its antitoxin partner Phd through fold complementation J Biol Chem 2008 283 45 30821 30827 10.1074/jbc.M805654200 18757857 PMC2576525

[B26] Komano T Utsumi R Kawamukai M Functional analysis of the fic gene involved in regulation of cell division Res Microbiol 1991 142 2–3 269 277 1656497 10.1016/0923-2508(91)90040-h

[B27] Lehnherr H Maguin E Jafri S Yarmolinsky MB Plasmid addiction genes of bacteriophage P1: doc, which causes cell death on curing of prophage, and phd, which prevents host death when prophage is retained J Mol Biol 1993 233 3 414 428 10.1006/jmbi.1993.1521 8411153

[B28] Zekarias B Mattoo S Worby C Lehmann J Rosenbusch RF Corbeil LB *Histophilus somni* IbpA DR2/Fic in virulence and immunoprotection at the natural host alveolar epithelial barrier Infect Immun 2010 78 5 1850 1858 10.1128/IAI.01277-09 20176790 PMC2863524

[B29] Zielenkiewicz U Ceglowski P The toxin-antitoxin system of the streptococcal plasmid pSM19035 J Bacteriol 2005 187 17 6094 6105 10.1128/JB.187.17.6094-6105.2005 16109951 PMC1196172

[B30] Zhu WCN McDougal LK Hageman J McDonald LC Patel JB Vancomycin-resistant *Staphylococcus aureus* isolates associated with Inc18-like vanA plasmids in Michigan Antimicrob Agents Chemother 2008 52 452 457 10.1128/AAC.00908-07 18056272 PMC2224762

[B31] Brown JSGS Spratt BG Holden DW A locus contained within a variable region of pneumococcal pathogenicity island 1 contributes to virulence in mice Infect Immun 2004 72 1587 1593 10.1128/IAI.72.3.1587-1593.2004 14977965 PMC356060

[B32] Chen C Tang J Dong W Wang C Feng Y Wang J Zheng F Pan X Liu D Li M A glimpse of streptococcal toxic shock syndrome from comparative genomics of *S. suis* 2 Chinese isolates PLoS One 2007 2 3 e315 10.1371/journal.pone.0000315 17375201 PMC1820848

[B33] Meinhart A Alonso JC Strater N Saenger W Crystal structure of the plasmid maintenance system epsilon/zeta: functional mechanism of toxin zeta and inactivation by epsilon 2 zeta 2 complex formation Proc Natl Acad Sci USA 2003 100 4 1661 1666 10.1073/pnas.0434325100 12571357 PMC149889

[B34] Lioy VSMM Camacho AG Lurz R Antelmann H pSM19035-encoded zeta toxin induces stasis followed by death in a subpopulation of cells Microbiology 2006 152 2365 2379 10.1099/mic.0.28950-0 16849801

[B35] Kristoffersen P Jensen GB Gerdes K Piskur J Bacterial toxin–antitoxin gene system as a containment control in yeast cells Application Environment Microbiology 2000 66 5524 5526 10.1128/AEM.66.12.5524-5526.2000 PMC92497 11097943

[B36] Blower TR Salmond GPC Luisi B Balancing at survival's edge: the structure and adaptive benefits of prokaryotic toxin-antitoxin partners Curr Opin Struc Biol 2011 21 1 109 118 10.1016/j.sbi.2010.10.009 21315267

[B37] Bolhuis H Poele EM Rodriguez-Valera F Isolation and cultivation of Walsby's square archaeon Environ Microbiol 2004 6 12 1287 1291 10.1111/j.1462-2920.2004.00692.x 15560825

[B38] Christensen SK Pedersen K Hansen FG Gerdes K Toxin-antitoxin loci as stress-response-elements: ChpAK/MazF and ChpBK cleave translated RNAs and are counteracted by tmRNA J Mol Biol 2003 332 4 809 819 10.1016/S0022-2836(03)00922-7 12972253

[B39] Brocchi M de Vasconcelos ATR Zaha A Restriction-modification systems in *Mycoplasma spp* Genet Mol Biol 2007 30 1 236 244 10.1590/S1415-47572007000200011

[B40] Kobayashi I Behavior of restriction-modification systems as selfish mobile elements and their impact on genome evolution Nucleic Acids Res 2001 29 18 3742 3756 10.1093/nar/29.18.3742 11557807 PMC55917

[B41] Webb K Jolley KA Mitchell Z Robinson C Newton JR Maiden MC Waller A Development of an unambiguous and discriminatory multilocus sequence typing scheme for the *Streptococcus zooepidemicus* group Microbiology 2008 154 Pt 10 3016 3024 18832307 10.1099/mic.0.2008/018911-0

[B42] Katz ME Strugnell RA Rood JI Molecular characterization of a genomic region associated with virulence in *Dichelobacter nodosu*s Infect Immun 1992 60 11 4586 4592 1398971 10.1128/iai.60.11.4586-4592.1992 PMC258206

[B43] Bloomfield GA Whittle G McDonagh MB Katz ME Cheetham BF Analysis of sequences flanking the vap regions of *Dichelobacter nodosus*: evidence for multiple integration events, a killer system, and a new genetic element Microbiology 1997 143 Pt 2 553 562 9043132 10.1099/00221287-143-2-553

[B44] Romero P Croucher NJ Hiller NL Hu FZ Ehrlich GD Bentley SD Garcia E Mitchell TJ Comparative genomic analysis of ten *Streptococcus pneumoniae* temperate bacteriophages J Bacteriol 2009 191 15 4854 4862 10.1128/JB.01272-08 19502408 PMC2715734

[B45] Lindsay JA Ruzin A Ross HF Kurepina N Novick RP The gene for toxic shock toxin is carried by a family of mobile pathogenicity islands in *Staphylococcus aureus* Mol Microbiol 1998 29 2 527 543 10.1046/j.1365-2958.1998.00947.x 9720870

[B46] Ozeri V Rosenshine I Mosher DF Fassler R Hanski E Roles of integrins and fibronectin in the entry of *Streptococcus pyogenes* into cells via protein F1 Mol Microbiol 1998 30 3 625 637 10.1046/j.1365-2958.1998.01097.x 9822827

[B47] Schwarz-Linek U Hook M Potts JR The molecular basis of fibronectin-mediated bacterial adherence to host cells Mol Microbiol 2004 52 3 631 641 10.1111/j.1365-2958.2004.04027.x 15101971

[B48] Lannergard J Flock M Johansson S Flock JI Guss B Studies of fibronectin-binding proteins of *Streptococcus equi* Infect Immun 2005 73 11 7243 7251 10.1128/IAI.73.11.7243-7251.2005 16239519 PMC1273847

[B49] Telford JL Barocchi MA Margarit I Rappuoli R Grandi G Pili in gram-positive pathogens Nat Rev Microbiol 2006 4 7 509 519 10.1038/nrmicro1443 16778837

[B50] Wilson RL Elthon J Clegg S Jones BD *Salmonella enterica* serovars gallinarum and pullorum expressing *Salmonella enterica* serovar typhimurium type 1 fimbriae exhibit increased invasiveness for mammalian cells Infect Immun 2000 68 8 4782 4785 10.1128/IAI.68.8.4782-4785.2000 10899888 PMC98437

[B51] Ma Z Fan HJ Lu CP Molecular cloning and analysis of the UDP-Glucose Pyrophosphorylase in *Streptococcus equi* subsp. *zooepidemicus* Mol Biol Rep 2011 38 4 2751 2760 10.1007/s11033-010-0420-8 21104023

[B52] Takahashi Y Yoshida A Nagata E Hoshino T Oho T Awano S Takehara T Ansai T *Streptococcus anginosus* l-cysteine desulfhydrase gene expression is associated with abscess formation in BALB/c mice Mol Oral Microbiol 2011 26 3 221 227 10.1111/j.2041-1014.2010.00599.x 21545699

[B53] Gao F Zhang CT Ori-Finder: a web-based system for finding oriCs in unannotated bacterial genomes BMC Bioinforma 2008 9 79 10.1186/1471-2105-9-79 PMC2275245 18237442

[B54] Delcher AL Harmon D Kasif S White O Salzberg SL Improved microbial gene identification with GLIMMER Nucleic Acids Res 1999 27 23 4636 4641 10.1093/nar/27.23.4636 10556321 PMC148753

[B55] Tatusov RL Galperin MY Natale DA Koonin EV The COG database: a tool for genome-scale analysis of protein functions and evolution Nucleic Acids Res 2000 28 1 33 36 10.1093/nar/28.1.33 10592175 PMC102395

[B56] Mulder NJ Apweiler R The InterPro database and tools for protein domain analysis Curr Protoc Bioinformatics 2008 2 2 7 10.1002/0471250953.bi0207s21 18428686

[B57] Kanehisa M Goto S Hattori M Aoki-Kinoshita KF Itoh M Kawashima S Katayama T Araki M Hirakawa M From genomics to chemical genomics: new developments in KEGG Nucleic Acids Res 2006 34 D354 357 Database issue 10.1093/nar/gkj102 16381885 PMC1347464

[B58] Lowe TM Eddy SR tRNAscan-SE: a program for improved detection of transfer RNA genes in genomic sequence Nucleic Acids Res 1997 25 5 955 964 9023104 10.1093/nar/25.5.955 PMC146525

[B59] Lagesen K Hallin P Rodland EA Staerfeldt HH Rognes T Ussery DW RNAmmer: consistent and rapid annotation of ribosomal RNA genes Nucleic Acids Res 2007 35 9 3100 3108 10.1093/nar/gkm160 17452365 PMC1888812

[B60] Kurtz S Phillippy A Delcher AL Smoot M Shumway M Antonescu C Salzberg SL Versatile and open software for comparing large genomes Genome Biol 2004 5 2 R12 10.1186/gb-2004-5-2-r12 14759262 PMC395750

[B61] Alexeyenko A Tamas I Liu G Sonnhammer EL Automatic clustering of orthologs and inparalogs shared by multiple proteomes Bioinformatics 2006 22 14 e9 15 10.1093/bioinformatics/btl213 16873526

[B62] Thompson JD Gibson TJ Higgins DG Multiple sequence alignment using ClustalW and Clustal X Curr Protoc Bioinformatics 2002 2 2 3 18792934 10.1002/0471250953.bi0203s00

[B63] Tamura K Dudley J Nei M Kumar S MEGA4: Molecular Evolutionary Genetics Analysis (MEGA) software version 4.0 Mol Biol Evol 2007 24 8 1596 1599 10.1093/molbev/msm092 17488738

[B64] Carver TJ Rutherford KM Berriman M Rajandream MA Barrell BG Parkhill J ACT: the Artemis Comparison Tool Bioinformatics 2005 21 16 3422 3423 10.1093/bioinformatics/bti553 15976072

[B65] Mortazavi A Williams BA McCue K Schaeffer L Wold B Mapping and quantifying mammalian transcriptomes by RNA-Seq Nat Methods 2008 5 7 621 628 10.1038/nmeth.1226 18516045 PMC13303166

[B66] Romualdi C Bortoluzzi S D'Alessi F Danieli GA IDEG6: a web tool for detection of differentially expressed genes in multiple tag sampling experiments Physiol Genomics 2003 12 2 159 162 12429865 10.1152/physiolgenomics.00096.2002

[B67] Langille MG Brinkman FS IslandViewer: an integrated interface for computational identification and visualization of genomic islands Bioinformatics 2009 25 5 664 665 10.1093/bioinformatics/btp030 19151094 PMC2647836

[B68] Hong-Jie F Fu-yu T Ying M Cheng-ping L Virulence and antigenicity of the szp-gene deleted *Streptococcus equi* ssp. *zooepidemicus* mutant in mice Vaccine 2009 27 1 56 61 10.1016/j.vaccine.2008.10.037 18983882

